# Exploring teachers’ attitudes and self-efficacy beliefs for implementing student self-assessment of English as a foreign language writing

**DOI:** 10.3389/fpsyg.2022.956160

**Published:** 2022-08-25

**Authors:** Xiaoyu Sophia Zhang, Lawrence Jun Zhang, Judy M. Parr, Christine Biebricher

**Affiliations:** ^1^The Mind Lab, Master of Contemporary Education Programme, Auckland, New Zealand; ^2^Faculty of Education and Social Work, University of Auckland, Auckland, New Zealand

**Keywords:** teachers’ attitude, self-efficacy beliefs, self-assessment, English as a foreign language (EFL) writing, English language teaching

## Abstract

With the growing need to nurture students’ independent learning, English language teaching (ELT) practices should reflect student-centered assessment approaches, such as self-assessment, an ultimate goal of higher education. It has been pointed out that to conduct effective self-assessment, students need to be taught systematically, and that is where teachers are expected to step in. Prior to implementing such a change in ELT, it is important to conduct research on English as a foreign language (EFL) teachers’ attitudes toward, and self-efficacy beliefs about, implementing self-assessment to cultivate capable student self-assessors. Although the strong global endorsement of self-assessment over the past two decades has witnessed its classroom implementation in different disciplines, such studies are scant in relation to EFL writing classrooms. To address this gap, the present qualitative research examined five Chinese tertiary EFL writing teachers’ attitudes toward and self-efficacy beliefs about student self-assessment of writing, as well as possible reasons that discourage them from engaging students in self-assessment practices. Data collected from in-depth, semi-structured interviews indicated that self-assessment, a critical element of self-regulated learning, is surprisingly missing from the teachers’ knowledge base and previous practices. Additionally, the findings offer insights into the striking differences in teachers’ understanding of, attitudes toward, and low self-efficacy beliefs about self-assessment of writing. Reasons why teachers choose not to implement self-assessment of writing are also discussed. Findings from this study contribute to a deeper understanding of how EFL teachers’ attitudes and self-efficacy beliefs are enacted in relation to their classroom assessment practices in order to move forward discussions on the feasibility of implementing self-assessment of writing in tertiary EFL classrooms.

## Introduction

It has been increasingly acknowledged that the focus of higher education has shifted toward widening students’ engagement in learning so as to enhance students’ academic achievement (Dochy et al., 1999; Cassidy, 2007; Yan, 2020). There is a growing recognition of the value for teachers to implement Assessment for Learning (AfL), namely, a student-centered assessment approach, in classrooms globally (Birenbaum et al., 2015; Tai et al., 2018; Dixon et al., 2020). In the Chinese context, although AfL has been promoted across university classes at the policy level, for example, the Ministry of Education (MoE) of China has proposed and stressed AfL to be prioritized in every major curriculum in classroom practices (MoE P. R. China, 2017), EFL teaching and assessment are still mostly confined to an exam-driven, product-oriented, and teacher-dominated environment, particularly in the writing domain (see Berry, 2011; Brown and Gao, 2015; Reinders et al., 2017; Wu et al., 2021a; Yan et al., 2021). Therefore, how policies are enacted in EFL classroom assessment practices and how teachers conceive student-led assessment approaches are of significance and the latter is part of the current research endeavor.

Student-centered assessment approaches, such as self-assessment, afford students more opportunities to engage actively in their learning and assessment process. However, “a fine-tuned self-assessment ability does not come automatically to all students” (LeBlanc and Painchaud, 1985, p. 675; see also Gan, 2012; Reynolds et al., 2021; Wu et al., 2021c; Gan et al., 2022). That can only be developed with teachers’ support and empowerment throughout the learning journey (Sadler, 1989; Panadero et al., 2016a): first to raise awareness among learners of the benefits of self-assessment; second to provide guidance on, and materials for, conducting self-assessment; and third to help learners understand the significance of the results (Gardner, 2000). Though teachers play a salient role in nurturing students’ assessment capabilities, insufficient attention is paid to teachers’ beliefs and attitude toward assessment forms (Looney et al., 2018), particularly in the underexplored EFL context, in which the studies of how teachers have responded to student-centered assessment approaches in the writing domain are scarce in the existing literature (Liu and Xu, 2017; Wu et al., 2021b).

The study presented in this paper, as part of a larger study on teachers’ and students’ perceptions and experiences of self-assessment of writing, intends to address the above research gaps by scrutinizing five EFL teachers’ attitudes toward, and self-efficacy beliefs for implementing self-assessment of writing in their classrooms using in-depth interviews. How EFL teachers in Chinese universities perceive self-assessment of writing and how self-efficacious they are to implement self-assessment practices are questions that urgently need investigation because teachers’ perceptions and self-efficacy beliefs could greatly influence the assessment approaches (Looney et al., 2018) and the quality of language education in Chinese tertiary settings. Further, it is expected that findings of this study could help English writing teachers understand the current uptake of self-assessment practices and, extrapolating from these data, we argue that EFL teachers’ assessment literacy development serves as a prerequisite for successful implementation of student-centered assessment in classrooms (Mohammadkhah et al., 2022; Tajeddin et al., 2022;).

## Literature review

### Self-assessment and English as a foreign language writing

Self-assessment, within which terms such as self-marking, self-revision, and self-evaluation are included (Sadek, 2018), is a nebulous term lacking a uniform definition due to its complexity and variability (Andrade, 2019). One of the widely accepted definitions refers to self-assessment as a “wide variety of mechanisms and techniques through which students describe (i.e., assess) and possibly assign merit or worth to (i.e., evaluate) the qualities of their own learning processes and products” (Panadero et al., 2016b, p. 804). Such a definition has attached a learning-oriented purpose to self-assessment (Andrade, 2010, 2019; Huang, 2016; Yan, 2018), and therefore many scholars have differentiated formative self-assessment from summative assessment (Panadero et al., 2016b; Van Reybroeck et al., 2017) given that writing is an important means by which learners acquire foreign language skills (Zhang, 2022).

In a formative sense, self-assessment is conceptualized as a cyclical learning process (Black and Wiliam, 1998; Andrade and Du, 2007; McMillan and Hearn, 2008; Yan, 2018), “during which students collect information about their own performance, evaluate and reflect on the quality of their learning process model and outcomes according to selected criteria, to identify their own strengths and weaknesses” (Yan and Brown, 2017, p. 2). In that learning process, a wide range of exercises such as self-revising/reflection, performance estimation, criteria-or rubric-based assessments are included. On the other hand, summative self-assessment implies that it is used as a measurement tool by teachers to empower and encourage students to simply rate, or grade, their final work to understand their proficiency in a certain task and to improve their performance (Boud, 1995; Butler and Lee, 2010; Pinner, 2016; Harris and Brown, 2018). Though summative self-assessment practices may pressure students to make a judgment, they could be a useful starting point and supplement for students to develop realistic and sophisticated formative self-assessment (Brown and Harris, 2014; Harris and Brown, 2018). The current study aims to examine teachers’ attitude and self-efficacy beliefs for both types of self-assessment as a mechanism for students to assess, to analyze and to improve their writing performance.

Over the last three decades, a substantial body of research has explored the close connections between self-assessment and writing from three key areas, namely, the accuracy of students’ self-grading in relation to that of the teacher’s grading, the effect of using self-assessment approaches on students’ overall writing, and student’ and teachers’ attitudes toward and experiences in self-assessment of writing (e.g., Boud, 1989; Sadler, 1989; Nielsen, 2012; Burner, 2016; Lam, 2016; Wang, 2016; Lee, 2017; Graham and Alves, 2021; Takrouni and Assalahi, 2022). However, this research field is still in its infancy due to the lack of consideration of self-assessment at different levels of education in non-western countries (for reviews, see Riazi et al., 2018; Zheng and Yu, 2019) and the possible tension it creates in “teacher-centered” learning contexts (Carless, 2011). With little attention paid to EFL writing in early research on self-assessment, there has been an increase in the number of studies in Asian and African countries, especially in recent years during the Pandemic COVID-19 (e.g., Bouziane and Zyad, 2018; Ghazizadeh and Taghipour Bazargani, 2019; Liu and Brantmeier, 2019; Mat and Par, 2022; Rezai et al., 2022; Takrouni and Assalahi, 2022). Some of those studies have investigated the effects of implementing self-assessment in the EFL writing class and have reported significant benefits of such an approach (Matsuno, 2009; Birjandi and Siyyari, 2010; Birjandi and Hadidi Tamjid, 2012). However, since those studies tended to integrate self-assessment together with peer-assessment and teacher-assessment, their mixed findings were not helpful in identifying the effects of implementing self-assessment alone. A recent study from Mazloomi and Khabiri (2018) has made an effort to fill that gap by using only self-assessment as an intervention, aiming to improve students’ writing skills and language proficiency; their findings echoed the research conducted by Mok et al. (2006), indicating that teachers’ appropriate feedback and students’ continuous engagement in self-assessment serve as the prerequisites for EFL writing improvement (Yu, 2021; Zhang and Cheng, 2021). Teachers’ perceptions of implementing student self-assessment of writing at the tertiary level have also been considered, with challenges in self-assessment integration identified in two major categories, namely, students’ personal capability, and affordance within workplace context (Takrouni and Assalahi, 2022).

Also examining the use of self-assessment in the writing domain, a small group of studies in China concern young learners in the classroom. These studies have been conducted mostly in Hong Kong, which is a different context compared to mainland China due to its education system and learning assessment culture having been influenced by British traditions (Bai et al., 2019). With young learners, Lee and her colleagues (Lee, 2011a,b; Lee and Coniam, 2013) have used self- and peer-assessment with writing in their case studies of secondary students and teachers. Their studies only implemented self-assessment in a limited form, for example, students self-edited their writing according to the prepared checklists, an approach which, arguably, was not sufficient to engage students fully in self-assessing their writing processes and products. However, those studies have indicated both the favorable effects of self-assessment on students’ learning attitudes and writing performance and teachers’ dilemmas and challenges when confronted with these assessment approaches. Such findings were echoed in Lam’s (2013) study, where the link between self-assessment and text-revision was shown. More recently, in mainland China, where significant correlations were found between self-assessment of reading/writing scores and reading comprehension/writing test scores, Liu and Brantmeier’s (2019) study has demonstrated that Chinese young learners could self-assess their English reading/writing skills and knowledge quite accurately. Nevertheless, further details are needed not only regarding the processes that young learners use to self-assess their reading and writing, but also how language teachers’ assessment literacy can be further developed to support students’ growing needs in such assessment activities (Yan et al., 2021).

With respect to the limited number of studies that have focused on university level students (e.g., Liu, 2002; Zheng et al., 2007; Wang, 2016; Wu et al., 2021c), Liu’s (2002) study appears to be the first one using self-assessment in the English writing classes. Findings of Liu’s study suggested that Chinese university students’ self-assessment reliability appeared to be affected by students’ English proficiency. However, such findings could be problematic since Liu simply defined English major students as high proficiency and Japanese major students as low proficiency, and students from the two groups self-assessed essays on different topics. Similar to the L1 context, some Chinese researchers have investigated the effectiveness of rubric-referenced self-assessment in writing classrooms (e.g., Zheng et al., 2007; Wang, 2016; Xu, 2019). Unlike Liu’s (2002) study, these studies mostly involved training sessions to help students to understand and use the rubric. The value of using a rubric to develop students’ capabilities in self-assessing writing was affirmed by students’ growth in writing performance, self-assessment accuracy, and self-efficacy. While research into peer feedback has also been reported, unlike what has been observed by Sadler (1989), namely, that peer-assessment serves as a preliminary step for self-assessment, often peer feedback has not been researched as an essential part of student self-assessment to promote EFL writing development.

In sum, though previous studies employing self-assessment in the EFL writing class have shown its positive effects on students’ writing performance, very few have examined teachers’ attitudes and self-efficacy levels toward the implementation of self-assessment and this study intends to fill in that gap.

### English as a foreign language teachers’ attitudes toward self-assessment

Teachers’ perceptions of assessment practices are deemed as significant indicators of their assessment literacy and assessment approaches (Looney et al., 2018; Wu et al., 2021c). Further, teachers’ initiatives and attitudes toward self-assessment are closely associated with the growth of student self-assessment capabilities (Tan, 2007; Bourke, 2014). However, compared to the relatively large body of literature on teachers’ attitudes toward peer-assessment (see Chang, 2016; Zhang and Cheng, 2020, for a review), existing studies investigating self-assessment from the EFL teachers’ perspective are rare, with more research in this avenue seeming to emerge in recent years (Maxfield, 2022). For example, in studies conducted in Sweden, Norway and Saudi Arabia, secondary and university students’ and teachers experiences of implementing self-assessment into every day writing practices were explored (Oscarson, 2009; Burner, 2016; Takrouni and Assalahi, 2022). In those studies, teachers acknowledged the considerable benefits for students of using self-assessment, such as providing teachers insights into the difficulties that students may face in learning and allowing teachers to improve teaching practice based on student self-assessment (Maxfield, 2022). However, teachers were too conservative to accept student self-assessment and integrate it in their writing classroom.

Regarding the Chinese context, Brown and Gao (2015) synthesized recent studies of how practicing teachers conceived the nature and purposes of assessment. They identified that teachers hold conflicting conceptions of assessment, in other words, Chinese teachers prefer a student-centered and learning-oriented assessment, yet they do not trust it and they need more professional development sessions on how to implement it; their conception may also be affected by occasions and locations. Similar results were also obtained from recent studies (Wu et al., 2021a,c), in which a quantitative and a qualitative research design was adopted, respectively, to explore how EFL teachers in Chinese universities perceive and utilize AfL strategies in their classroom. In the quantitative study (Wu et al., 2021a), the 402 respondent teachers showed great reluctance in implementing student-led assessments (self-assessment and peer-assessment) even though they tended to attach value to such practice. Further, in the qualitative case studies, three teacher participants demonstrated their diverse assessment practices and attitudes toward student-led assessment approaches in their writing classrooms.

### Teachers’ self-efficacy beliefs for assessment

Following social cognitive theory, self-efficacy refers to “…beliefs in one’s capabilities to organize and execute the courses of action required producing given attainments” (Bandura, 1997, p. 3). Specifically, in educational contexts, teacher efficacy is considered as a domain of self-efficacy, which could be defined as teachers’ “individual beliefs in their capabilities to perform specific teaching tasks at a specified level of quality in a specified situation” (Dellinger et al., 2008, p. 752). Students’ self-efficacy plays a key role in their behavior and academic performance (Noorollahi, 2021), whereas teachers’ self-efficacy beliefs are crucial not only in shaping how much they influence students’ academic outcome through their activity choices (Klassen et al., 2009; Zee and Koomen, 2016; Wyatt, 2018), but also in predicting their use of formative assessment practices (Thompson, 2020; Xiang et al., 2020). For example, teachers with higher self-efficacy beliefs are less resistant to novel ideas and methods (Tschannen-Moran and Hoy, 2001) Therefore, they are more likely to invest significant time and effort to experiment with student-centered assessment approaches and support their students with enthusiasm in performing self-assessment or peer-assessment than those who are less efficacious (Bandura, 1997; Hoang and Wyatt, 2021).

Previous studies have examined the effects of teacher efficacy in a range of dimensions, such as instruction, classroom management, student engagement, and collegial collaboration (Chan, 2008a,b; Klassen et al., 2009; Poulou et al., 2019), and despite the fact that research into self-efficacy beliefs of EFL teachers is a burgeoning field in recent years (Hoang, 2018; Wyatt, 2018; Thompson, 2020), teachers’ self-efficacy beliefs in assessment practices are rarely explored. Similarly, Malinen’s (2016) review with respect to the trends, research questions and instruments in existing empirical studies concerning teacher self-efficacy in mainland China has pointed out the ongoing scarcity of such research in international literature, particularly for research written in the English language. The reason why this type of research is not common in Mainland China might be that the majority of the research instruments of self-efficacy originated in the west, and they have not yet been adapted sufficiently for Confucian-heritage cultures, nor have they designed their own (Hoang and Wyatt, 2021).

The available research into EFL teachers’ attitude toward and self-efficacy beliefs for assessment, despite its paucity, provides insights for further research.

Contextualizing within the Chinese tertiary language teaching setting, this study is motivated and guided by the following two research questions:

(1) What are EFL writing teachers’ attitudes toward and perceptions of student self-assessment of writing?
(2) How self-efficacious are EFL writing teachers in implementing self-assessment of writing?

Through answering the above questions, the present study also aims to explore the primary reasons for the underuse of self-assessment of writing among tertiary EFL teachers in China.

## Materials and methods

As a qualitative inquiry framed within the theory of constructivism, this study was carried out aiming to understand the attitude, self-efficacy beliefs, and practices of self-assessment of writing that EFL teachers construct from their own experience and social cultural context.

### Participants

#### Sampling strategy

In the present research, the participating teachers were selected through convenience sampling, which is “selecting a sample based on time, money, location, availability of sites or respondents, and so on” (Merriam and Tisdell, 2016, p. 98). We contacted potential EFL writing teachers from several universities in a Northern Chinese city and, after initial discussions, five female teachers from two medium sized universities (with 12,000–20,000 students) agreed to participate in this research as it interested them. The five participants are part of a majority of the EFL teacher population in China as, according to the statistics provided by Ministry of Education of the People’s Republic of China (2020), females make up more than half of tertiary EFL teachers. An information sheet with detailed explanation of the research purpose and processes, as well as ethical considerations and a consent letter were provided to the participants prior to the interviews. In the following section, the participants’ backgrounds are described.

#### Participant background

Table 1 demonstrates the participants’ demographic information overview, and the participants are referred to as Yvonne, Grace, Sue, Wendy and Lee, which are not their real names. We do so for personal data protection.

**TABLE 1:** Overview of participants’ demographics.

Name	Gender	Age	Educational degree	Years of teaching	Course	Students’ university year level
Yvonne	Female	33	Master of arts	8	English writing	3
Grace	Female	43	Master of arts	19	English writing	3
Sue	Female	31	Master of arts	5	Essay writing	2
Wendy	Female	38	Master of arts	15	Academic writing	4
Lee	Female	26	Master of arts	2	Essay writing	2

As indicated in Table 1, though the five participants share similar educational backgrounds and are teaching parallel English writing courses, they vary considerably in their ages, stages of careers (years of teaching experience), and students’ year levels taught (students in levels 2, 3, 4 refer to sophomores, juniors, and seniors, respectively). While the similarities among the participants help to generate a plausible picture of EFL writing teachers’ attitudes and self-efficacy beliefs in self-assessment of writing in the region, their differences allow a range of views to be represented (Creswell and Creswell, 2018).

### Interviewing as a research tool

Carried out in varied forms (e.g., telephone, focused groups, and semi-structured interviews), interviews are frequently adopted as a powerful data collection instrument in educational research for in-depth investigation (Creswell and Creswell, 2018), especially when direct observation of participants’ behavior, feelings and interpretation of the world around them is not feasible (Sharan and Tisdell, 2016). During interviews, a number of open-ended questions are generally asked to elicit participants’ opinions and to unfold participants’ experiences in diverse topics (Adhabi and Anozie, 2017). Interviews also allow flexibility for both the interviewer and the interviewees. On the one hand, in interviews the interview protocol can be adapted to various situations regarding the line of questioning and, on the other hand, interviewees are able to explain newly emerged issues so that a systematic coverage of the targeted domain is ensured (Rubin and Rubin, 2011). Having considered the advantages of interviews, it is important to point out their caveats, which are mostly due to the possible power imbalances or language related issues existing between the researcher and the interviewees that may bias responses to different extent (Rolland et al., 2020).

Based on the amount of structure, one-to-one interviews are divided into three types, namely, structured, unstructured, and semi-structured. When placed on a continuum, structured and unstructured interviews are two extremes in which the interviewer either strictly adheres to a predetermined interview scheme or loosely follows the prepared interview schedule so that there is minimal interruption when interviewees bring out unpredictable issues (Sharan and Tisdell, 2016). In the middle position between structured and unstructured interviews, semi-structured interviews have witnessed great popularity in the applied linguistic domain because of theirs relative flexibility regarding the open-ended format in which the interviewer points out the main direction (the structured part) and the interviewees have room for “variation or spontaneity in responses” (the semi- part) to explicate more on certain issue to supplement major questions from various lens (Dörnyei, 2007, p. 136).

While limited in scope, for example, sample size and what is asked about, the present research aims to provide rich information about EFL writing teachers’ attitudes toward and self-efficacy beliefs about self-assessment of writing implementation; interviews, particularly semi-structured interviews, are appropriate to obtain verbal data to achieve the purpose of this study for three reasons. Initially, interview data help more than the questionnaire data to provide in-depth and vivid material from interviewees (Ritchie et al., 2014; Wyatt, 2018). Secondly, the direct contact with the interviewees not only allows the researcher to record both verbal and non-verbal data communications, but also to check for accuracy and relevance during the interview, which to some extent, ensures the data validity (Denscombe, 2014). Lastly, avoiding the drawbacks of both structured interviews and unstructured interviews, semi-structured interviews are optimal to facilitate the researcher and the interviewees reaching a mutual understanding of self-assessment of writing through conversational discussions (Zhao, 2018).

Interviews were conducted and directed by the first author with two broad open-ended questions below:

1) What do you think of student self-assessment of writing?
2) Out of 100, how confident are you to implement self-assessment of writing in your class? And why?

The first question was to reveal a realistic picture of interviewees’ basic understanding of and attitudes to student self-assessment of writing. It was intended to elucidate further discussions of interviewees’ self-efficacy in self-assessment of writing implementation and potential reasons for using or not using student self-assessment (Question 2). Even though all the participants viewed themselves as proficient English speakers, they chose to be interviewed in Mandarin for two reasons, namely, that using their mother tongue was not only helpful to express themselves in a deeper sense regarding matters pertinent to English teaching and assessment practices during the interview, but also it brought the interviewer and interviewee closer for open discussions and honest answers concerning the topic, to avoid ambiguity.

### Data collection and analysis procedures

For the current study, the data were collected through individual face-to-face interviews in a quiet setting in the participants’ respective universities over the course of a month, during which teachers tried out self-assessment with their students (Wu et al., 2021b). Each informant was interviewed once and each interview lasted around 1 h and a half generating rich data. The interview data were in the form of audio clips, and the first author also took field notes during and after the interviews.

Upon the completion of the last interview, data analysis procedures began. Firstly, the audio recordings, collected over the five interviews, were transcribed verbatim; secondly, the interviewer’s field notes taken during and after each interview were typed and summarized. The data were then processed employing thematic analysis, which is a qualitative approach for researchers to capture, analyze, and report patterns (themes) and concepts emerged from datasets (Braun and Clarke, 2006; Rubin and Rubin, 2011; Braun et al., 2019). Specifically, I followed six phases that describe how the emerging themes could be identified, improved, and settled. Table 2 taken from Braun and Clarke (2006, p. 35), shows the six phases in detail.

**TABLE 2:** Six phases of thematic analysis.

Phase	Description of the process
1. Familiarizing yourself with your data	Transcribing data (if necessary), reading and re- reading the data, noting down initial ideas.
2. Generating initial codes:	Coding interesting features of the data in a systematic fashion across the entire data set, collating data relevant to each code.
3. Searching for themes:	Collating codes into potential themes, gathering all data relevant to each potential theme.
4. Reviewing themes	Checking in the themes work in relation to the coded extracts (Level 1) and the entire data set (Level 2), generating a thematic “map” of the analysis.
5. Defining and naming themes:	Ongoing analysis to refine the specifics of each theme, and the overall story the analysis tells; generating clear definitions and names for each theme.
6. Producing the report	The final opportunity for analysis. Selection of vivid, compelling extract examples, the final analysis of selected extracts, relating back of the analysis to the research question and literature, producing a scholarly report of the analysis.

To establish the trustworthiness of inference for the qualitative data, we followed the criteria introduced in Lincoln and Guba (1985), further elaborated in Nowell et al. (2017). In addition, while conducting the data analysis, we did not translate the non-English interview data nor the first analyses into English for two reasons. Initially, meanings could be better conveyed and understood between the interviewer and the interviewees using their first language—Mandarin. Further, it is argued that “the relation between subjective experience and language is a two-way process; language is used to express meaning, but the other way round, language influences how meaning is constructed” (Van Nes et al., 2010, p. 313–314); much meaning/information may be lost when translating the source language to the target language due to the lack of proper wordings and cultural knowledge. Therefore, by mostly following the original Chinese interview transcripts during analysis, the above-mentioned possible drawbacks could be avoided. Later, to ensure the data translation and interpretation authenticity, two bilinguals with high Mandarin and English proficiency were invited, with one translating the transcripts from English to Mandarin, and the other translating those transcripts back from Mandarin to English. Then, interview transcripts and translation, themes, and analyses were shared with the teacher participants for member checking to elaborate and adjust their responses so that possible discrepancies in our interpretations could be dealt with appropriately (Rallis and Rossman, 2009). The first author has also endeavored to provide detailed and faithful descriptions of participants’ responses to present the reported findings to a wider international audience better and to triangulate the informants’ self-reported data with current literature so as to increase the possibility of finding transferability in similar contexts (Creswell and Creswell, 2018).

## Findings and discussion

Salient points from the interviews were summarized and results are reported below according to the research questions with different themes under each question that emerged from interviewees’ responses.

### RQ1: What are the English as a foreign language writing teachers’ attitude toward and perceptions of student self-assessment of writing

The data suggested that most teachers hold limited understanding of self-assessment of writing, and they tend to conceptualize both summative and formative self-assessment as a “taster,” which is not a real assessment. The following dimensions have been identified from the participating teachers’ diverse perceptions of self-assessment of writing.

#### Benefits of practicing self-assessment

Generally, all participating teachers demonstrated favorable attitude toward teaching student self-assessment of writing. The five teachers saw the potential of self-assessment of writing in improving teaching practices and students’ learning from varied perspectives. Taking teaching practices as an example, Grace mentioned that students’ successful self-assessment could inform her if the lesson planning was effective or not. Where planning or the implementation was not successful, Sue pointed out that the results of student self-assessment might help identify the areas of instruction that should be emphasized. These findings are similar to those reported by Brown and Gao (2015), who noted that Chinese teachers were inclined to utilize assessment as an information source to make a diagnosis of their teaching effectiveness and to adjust teaching strategies and instructional approaches accordingly (Yu, 2021; Maxfield, 2022).

As reflected in Lee’s comments, self-assessment was helpful for students to identify their writing weaknesses, for example, the spelling mistakes, and structure issues. Similar to Lee’s view, Sue and Grace considered, self-assessment to be beneficial in making students accountable for their writing in the long run by revisiting the writing criteria, structure, logic, and expressions multiple times themselves, actions that will foster students’ self-regulation skills (Brown et al., 2011; Kim et al., 2015; Wang and Lee, 2021), which are transferrable to other aspects of learning (Brown and Gao, 2015). Yvonne spoke more explicitly about how the self-assessment process could help students digest the criteria and thereby develop self-regulation skills, and her comments supported evidence from previous classroom observations (e.g., Harris and Brown, 2013; Bourke, 2018).

Yvonne: While self-assessing their writing against the criteria, students get to understand their writing in a deeper sense in different dimensions because students usually do not read what they write again after they submit that to their teacher, there is then no self-reflection going on at all. In addition, self-checking the basics such as spelling, and vocabulary usage will help students improve accuracy by avoiding those mistakes in future writing tasks. Or if students could not self-assess certain dimensions, they’ll reach out to seek relevant information.

Furthermore, as Wendy’s final comments illustrated, self-assessment could be of great help to those students who feel uncomfortable about their peers or teachers viewing and judging their writing work. Self-assessment of writing, particularly against a rubric, somehow acts like a guide for such students to gain clarity regarding general writing expectations and, at the same time, helps student keep their writing private (Ghaffar et al., 2020).

#### Ineffectiveness of student self-assessment of writing

Despite teachers’ optimistic views about self-assessment of writing, they did not overlook its shortcomings. For example, while teachers noted that self-assessment could promote self-regulated learning and deeper thinking in EFL writing and other social conditions, they clearly harbored concerns about the issue of no guarantee of full student participation in self-assessment. Likewise, emerging from the data, self-assessment of writing was not considered as the most effective assessment approach in terms of student engagement when compared with peer-assessment due to the limited levels of learner involvement in relatively isolated self-assessment tasks/activities (Gardner, 2000; Wanner and Palmer, 2018; Rezai et al., 2022). Wendy illustrated, in detail, her idea about self-assessment in comparison with peer-assessment, and that accorded with Sadler’s (1989) earlier observations, which showed that self-assessment is harder than peer assessment, so often peer assessment is an initial step.

Wendy: Peer-assessment introduces a sense of competition in the classroom so that students could easily participate more in the learning process, and it livens up the atmosphere. But it seems self-assessment only involves oneself; I then think self-assessment is quite boring for students and it is more difficult than peer-assessment for students to practice and for practitioners to achieve an active class. Without teachers’ help, it’s also difficult for students to know the quality of their self-assessment.

Similarly, echoing Harris and Brown’s (2013) findings in the New Zealand context, Yvonne explained that her understanding of the drawbacks of self-assessment of writing was pertinent to unbalanced teacher-student, and insufficient student-student, interaction (Orona et al., 2022).

Yvonne: Self-assessment of writing has its weaknesses in providing in-time instantaneous teacher-student interaction. It is difficult for us to notice and monitor individual student’s needs and progress unless they reach out for specific help during self-assessment, so that our attention to students could be imbalanced and that will bring negative effects on our teaching efficiency. Also, I guess in-class student-student interactions can be constrained by self-assessment due to its relatively isolated form, and that is not good for students’ oral skills development either.

Yvonne’s negative view of self-assessment reflected her uncertainty regarding the approach and the extent of teacher participation/facilitation in the self-assessment process, and her thoughts were in agreement with Lee’s articulation that self-assessment relied heavily on students themselves, who were not accustomed to take full responsibility for their own learning. Grace also mentioned that in self-assessment, the role of the students changed from traditional knowledge receivers to the owners of their learning, and such radical transition may cast a negative impact on students’ learning because students, as inexperienced self-assessors, may not be able to source effective supplementary materials to support their writing needs. Further, as inexperienced self-assessors. Students may not able to provide truthful and constructive feedback for themselves (Earl and Katz, 2006; Sambell et al., 2013). Therefore, teachers should be the sole source of feedback for students and self-assessment is unlikely to be effective in improving students’ writing performance in the short term (Brown and Gao, 2015).

In summary, the above findings are consistent with previous studies (Lee, 2011a,b; Lee and Coniam, 2013; Brown and Gao, 2015), indicating that though the participating teachers acknowledged the range of positive impacts that self-assessment has on students’ learning, they still preferred more traditional teacher-controlled assessments to fast track the learning progress, a belief reinforced by the university’s constant summative grading practices (Oscarson, 2009; Burner, 2016; Wu et al., 2021c). These teachers’ perceptions have also revealed somewhat stereotypical ideas about self-assessment and a neglect of the potential benefits to empower students to be responsible of their learning (Lee, 2016; Wang and Lee, 2021; Wu et al., 2021a).

### RQ2: How self-efficacious are English as a foreign language writing teachers in implementing self-assessment of writing?

During the past fifteen years, worldwide research in language teachers’ efficacy beliefs has indicated that teachers who are less self-efficacious are likely to be resistant to novel activities that are considered as helpful for language learning (Hoang and Wyatt, 2021). Findings in this research have corroborated that idea because all the participating teachers rated themselves relatively low in self-efficacy levels in terms of their confidence to implement self-assessment of writing (Ranging from 20 to 35 out of 100), either in a summative or formative sense. The reasons why teachers were not confident in implementing self-assessment of writing were mostly due to their lack of experience of such a student-centered assessment approach and the lack of relevant knowledge.

#### Reasons for teachers’ low self-efficacy beliefs for implementing self-assessment of writing

In accordance with the previous findings, the current study suggests that teachers’ lack of previous experiences of implementing self-assessment of writing could be one of the main reasons that they are not willing to go against the traditional teacher assessment path (Oscarson, 2009; Burner, 2016). Consistent with Wu et al. (2021a)’s survey results, which have reported Chinese university EFL teachers’ indifference to the value of student-centered assessment approaches and rare usage of peer- and self-assessment in classrooms, four out of five participating teachers (the exception was Lee) in our study reflected on their English teaching experiences and indicated that they have never used self-assessment in any form to evaluate their students’ writing or other aspects of English learning; they were not familiar with any forms of self-assessment, and not sure what kind of feedback should be provided for student self-assessment of writing either. Mostly, those teachers preferred using and emphasizing summative tasks (tests, essays, projects) to evaluate students’ writing performance against certain criteria (Lee, 2016; Wu et al., 2021c; Yan et al., 2021) as providing individualized feedback based on students’ self-assessment of writing is deemed as a challenging task in the Chinese EFL context (Yu, 2021). The following excerpts are examples of the common thoughts:

Yvonne: Asking students to self-assess their writing has never occurred to my mind. If they self-assess, what should I do? Do I still have control over the classroom?

Wendy: Self-assessment is just not in our assessment choices list; we somehow tend to ignore it as we don’t have much time for students to consider that properly. That’s too time consuming.

Grace: Student self-assessment is not in the teaching syllabus that we are asked to follow for each lesson, so why bother to do that then?

Sue: I guess summative tasks give us better sense of security to achieve a higher grade for students, and self-assessment doesn’t seem to fit in our learning culture. We don’t encourage any form of individualism here, by that I meant self-assessment somehow falls into that category.

The only exception to these views, Lee, was, comparatively, a younger teacher than other participants, and she explained that she had previously used limited self-assessment activities in an informal manner. There were no grade records, and she did not spend time checking if students had truly engaged in the self-assessment or not. Lee illustrated more details in her quote below.

Lee: When a semester ends, I’ll ask students to reflect on their general writing performance over that term and write a short self-reflection essay but that is not mandatory and no students have ever submitted such essays to me; I’ve also asked students to check their grammar mistakes by using a grammar checking software, I assume that can be counted as self-assessment?

These findings are broadly consistent with previous research into the sources of teacher self-efficacy, one of which, teachers’ accumulated mastery experience, has been identified as the most influential in self-efficacy enhancement (Bandura, 1997; Schunk and DiBenedetto, 2016). In the current study, student self-assessment is considered time-consuming (Lam, 2016) because even though AfL is promoted on a policy level, in reality, teaching in the traditional Chinese EFL writing classroom environment is driven mostly by frequent exams (Lee, 2014; Li et al., 2020) and teachers are accustomed to being proactive to manage their students’ writing tasks (Liu, 2002). Therefore, teachers’ low levels of self-efficacy for implementing self-assessment of writing might be explained by three reasons: their lack of knowledge in self-assessment; lack of opportunities to practice; and positive social persuasion (e.g., verbal or written encouragement and support from other colleagues, and from the university and the national policy), of which would serve as important influences that motivate teachers to use student self-assessment in their writing classes (Canbulat, 2017; Xiang et al., 2020; Takrouni and Assalahi, 2022). Further, the qualitative findings of this study are congruent with the few previous studies that identify teacher self-efficacy as a significant predictor of their usage of formative assessment practices (Thompson, 2020; Xiang et al., 2020) and favorable social conditions are the prerequisites for teachers to facilitate their students in cognitively demanding tasks such as self-assessment (Gan, 2012).

#### Challenges facing English as a foreign language teachers’ self-assessment of writing implementation

With low self-efficacy levels of implementing self-assessment of writing, the participating Chinese EFL teachers have signified several challenges, which are in accordance with those that have been identified by previous studies (Harris and Brown, 2013; Harris et al., 2018; Takrouni and Assalahi, 2022). Firstly, there is the challenge of overcoming the entrenched mind-set that assessing student writing is the teachers’ sole responsibility and teachers should be students’ main source of feedback for better exam results. Because of such a mindset, self-assessment is usually disregarded and believed to be less important compared with teacher-assessment, which in more compatible with the examination-oriented education system to bring students the expected outcomes in due course (Lee, 2011b; Harris et al., 2018; Takrouni and Assalahi, 2022). In the following interview quotes, Sue and Lee voiced their deep concern about the potential incompatibility of self-assessment with the current exam culture, indicating that if student self-assessment of writing cannot help students to achieve their academic goals in writing in the short term, teachers and students may both become demotivated, and they would soon shift their focus to traditional methods of assessment.

Sue: To help students improve their English writing to pass the national English tests is already difficult within the limited class time, not to mention introducing a new concept—self-assessment to writing. It will definitely take longer time for students to get used to it, and not to mention for bigger classes. We just can’t afford the time because we need to use that time wisely for exam preparation, and it seems teacher-assessment is the most effective way to help students.

Lee: I strongly doubt how helpful self-assessment would be to improve students’ English writing, because in the end, for both students and us, it is passing the exams that matters to all. You know, as a young teacher, every minute in class is precious, and I don’t want to waste the time to experiment if it’d be better to implement self-assessment of writing.

As seen in the above excerpts, the time-consuming nature of self-assessment makes it unlikely for Chinese EFL practitioners to achieve a balance between affording students enough independence to experience a sense of control, and offering them sufficient guidance and feedback in self-assessment writing procedures for them to achieve their desired academic goals (Andrade, 2010; Meihami and Razmjoo, 2016), particularly with a large number of learners in EFL classrooms (Lee, 2011a; Liu and Xu, 2017).

The second challenge concerns teachers’ lack of assessment literacy, including a limited understanding of assessment purposes and value, and mastery of assessment knowledge, which leads to teachers’ uncertainties about how to enact self-assessment appropriately (Mohammadkhah et al., 2022). If teachers implement self-assessment without sufficient relevant knowledge and concrete instruction, the practice might be superficial and not lead to students’ improvement (Panadero et al., 2016a). Supporting Andrade’s (2010) as well as Takrouni and Assalahi (2022) findings, in the following excerpt, Grace and Yvonne mentioned that their source of concern about self-assessment implementation mainly came from the insufficient professional development or faculty support in such areas.

Grace: How long should I assign to self-assessment of writing tasks for every class and for how long should I spend with each student to discuss his/her self-assessment of writing? My colleagues and I have never attended any professional development workshops focusing on that in my teaching career and I cannot even imagine scaffolding students’ self-assessment and teaching them the regular content and skills at the same time. How can we prepare the lesson plan together if none of us understands self-assessment properly? I don’t think students could self-assess their writing effectively when their teachers are not well informed about the practice of self-assessment.

Yvonne: There are usually more than 30 students in a class, what if I am not able to provide feedback on every student’s self-assessment of writing? If I continue talking to them after class, my workload will be significantly increased, and the faculty won’t recognize and support that. There are just too many questions unsolved if using an assessment approach that is not widely approved across the faculty.

Another challenge is that even though the participating teachers usually teach English major students, they firmly believed that those students’ self-assessing capabilities were rather low due to the fact that English is still their foreign language, in which the script, syllabus, and grammar could be different and difficult for them in a cognitive demanding task like self-assessment (Oscarson, 2009; Burner, 2016). The above excerpts are similar to those of other findings which indicate how the teacher’s lack of assessment literacy and organizational support is closely associated with whether teachers are able to work collaboratively with their colleagues to design context-specific self-assessment practices and whether they believe students could conduct self-assessment effectively (Huang and Luo, 2014; Andrade and Brookhart, 2016; Yan et al., 2019; Takrouni and Assalahi, 2022).

In view of the above multiple challenges, it seems that the participating teachers, regardless of the length of their teaching career, have not indicated a readiness to implement student self-assessment of writing in their classrooms. Student-centered assessment approaches have been advocated on the national policy level, however, our findings highlight teachers’ need of intensive professional support from their universities to improve their assessment literacy (Mohammadkhah et al., 2022; Takrouni and Assalahi, 2022), for example, getting familiar with the rationale and design of self-assessment activities so that their confidence in implementing self-assessment in the writing class could be enhanced (Wu et al., 2021a).

## Conclusion

Overall, although the current qualitative inquiry is small-scale and focused on female EFL writing teachers only, it is likely that those teachers’ attitudes and self-efficacy beliefs mirror the views and extent of implementation of self-assessment by other teachers elsewhere in China or other Asian contexts. Taken together, the research findings have not only indicated the disconnect between the Chinese national curriculum requirements and the reality of teaching practice, but also revealed theoretical and practical implications for EFL teachers. Theoretically, the current research has expanded, to a modest extent, the knowledge base and provided empirical evidence about EFL teachers’ attitudes toward and self-efficacy beliefs for self-assessment of writing from a qualitative perspective. Future research that uses questionnaires with robust construct and cultural validity will further help understand teacher self-efficacy in specific domains like this (Canbulat, 2017; Thompson, 2020).

Practically, findings from the present study have firstly pointed out the necessity to investigate further EFL teachers’ attitude and self-efficacy beliefs regarding student-led assessment approaches. Then, it is imperative for institutions to promote a professional development program dealing with teacher assessment literacy so that more EFL teachers can be helped to become confident in supporting their students to perform self-assessment of writing within their workload (Yu, 2021). Teachers’ capacity to perform student-led assessment approaches is contingent on “their access to high quality professional development activities designed to foster collaborative learning in interaction with other professionals in the area of assessment. Teachers’ new knowledge and skills will flourish, however, only if they fall on fertile ground” (Laveault and Allal, 2016, p. 15). Professional development in the area of assessment, therefore, is necessary to support teachers to up skill their knowledge in engaging students effectively in the complex self-assessment process, and to familiarize teachers with their varied roles and the different kinds of feedback they would provide for student self-assessment of writing (Wu et al., 2021a,b, c; Takrouni and Assalahi, 2022). Further, with much has been written regarding the challenges of AfL’s global implementation, this study adds to a clearer understanding of the underlying psychological reasons for the low application of self-assessment in EFL settings, namely, teachers’ uncertain attitudes and relatively low self-efficacy beliefs when they aspire to attempt to support student self -assessment practices.

Our study could have provided a more holistic view if we had engaged students’ perspectives along with those of their teachers’. Therefore, it is recommended for future research to take students’ viewpoints into consideration in conjunction with the teachers’ views, for instance, through interviews or learning journals (Rezai et al., 2022), so that data collected from students would provide feedback or rationale in terms of teachers’ attitude and self-efficacy beliefs in their classroom assessment practices. It is hoped that our findings can provide a reference, helping EFL teachers in similar educational cultures worldwide to understand, and to handle, the complexity of implementing student self-assessment in writing classrooms.

## Data availability statement

The original contributions presented in this study are included in the article/supplementary material, further inquiries can be directed to the corresponding author/s.

## Ethics statement

The studies involving human participants were reviewed and approved by the University of Auckland Human Ethics Committee. The patients/participants provided their written informed consent to participate in this study.

## Author contributions

XZ conceived contributions of the initial idea, fine-tuned by LZ, JP, and CB. XZ designed the study, collected and analyzed the data, and drafted of the manuscript. LZ, JP, and CB revised and proofread the manuscript. All authors agreed to the final version before LZ got it ready for submission as the corresponding author.
